# Vascular complications in patients with type 2 diabetes: prevalence and associated factors in 38 countries (the DISCOVER study program)

**DOI:** 10.1186/s12933-018-0787-8

**Published:** 2018-11-28

**Authors:** Mikhail Kosiborod, Marilia B. Gomes, Antonio Nicolucci, Stuart Pocock, Wolfgang Rathmann, Marina V. Shestakova, Hirotaka Watada, Iichiro Shimomura, Hungta Chen, Javier Cid-Ruzafa, Peter Fenici, Niklas Hammar, Filip Surmont, Fengming Tang, Kamlesh Khunti, for The DISCOVER investigators

**Affiliations:** 1 Saint Luke’s Mid America Heart Institute, 4401 Wornall Road, Kansas City, MO 64111 USA; 2 University of Missouri, Kansas City, 5100 Rockhill Rd, Kansas City, MO 64110 USA; 3 Rio de Janeiro State University, Av 28 de Setembro 77, Rio de Janeiro, CEP20555-030 Brazil; 4 Center for Outcomes Research and Clinical Epidemiology, Via Tiziano Vecellio, 2, 65124 Pescara, Italy; 5 London School of Hygiene and Tropical Medicine, Keppel St, Bloomsbury, London, WC1E 7HT UK; 6 Institute for Biometrics and Epidemiology, German Diabetes Center, Auf’m Hennekamp 65, 40225 Düsseldorf, Germany; 7 Endocrinology Research Center, Moskvorech’ye Ulitsa, 1, Moscow, 115478 Russian Federation; 8 Graduate School of Medicine, Juntendo University, 2-1-1 Hongo, Bunkyo-ku, Tokyo, 113-8421 Japan; 9 Graduate School of Medicine, Osaka University, 2-2 Yamadaoka, Suita, Osaka 565-0871 Japan; 10 AstraZeneca, 950 Wind River Ln, Gaithersburg, MD 20878 USA; 11 Evidera, Metro Building, 6th Floor, 1 Butterwick, London, W6 8DL UK; 12 AstraZeneca, 132 Hills Rd, Cambridge, CB2 1PG UK; 13 AstraZeneca Gothenburg, Pepparedsleden 1, 431 50 Mölndal, Sweden; 14 Institute of Environmental Medicine, Karolinska Institutet, Solnavägen 1, 171 77 Solna, Sweden; 15 AstraZeneca, 600 Capability Green, Luton, LU1 3LU UK; 16 University of Leicester, University Rd, Leicester, LE1 7RH UK

**Keywords:** Type 2 diabetes, Vascular complications, Observational study

## Abstract

**Background:**

The global prevalence of type 2 diabetes-related complications is not well described. We assessed prevalence of vascular complications at baseline in DISCOVER (NCT02322762; NCT02226822), a global, prospective, observational study program of 15,992 patients with type 2 diabetes initiating second-line therapy, conducted across 38 countries.

**Methods:**

Patients were recruited from primary and specialist healthcare settings. Data were collected using a standardized case report form. Prevalence estimates of microvascular and macrovascular complications at baseline were assessed overall and by country and region, and were standardized for age and sex. Modified Poisson regression was used to assess factors associated with the prevalence of complications.

**Results:**

The median duration of type 2 diabetes was 4.1 years (interquartile range [IQR]: 1.9–7.9 years), and the median glycated hemoglobin (HbA_1c_) level was 8.0% (IQR: 7.2–9.1%). The crude prevalences of microvascular and macrovascular complications were 18.8% and 12.7%, respectively. Common microvascular complications were peripheral neuropathy (7.7%), chronic kidney disease (5.0%), and albuminuria (4.3%). Common macrovascular complications were coronary artery disease (8.2%), heart failure (3.3%) and stroke (2.2%). The age- and sex-standardized prevalence of microvascular complications was 17.9% (95% confidence interval [CI] 17.3–18.6%), ranging from 14.2% in the Americas to 20.4% in Europe. The age- and sex-standardized prevalence of macrovascular complications was 9.2% (95% CI 8.7–9.7%), ranging from 4.1% in South-East Asia to 18.8% in Europe. Factors positively associated with vascular complications included age (per 10-year increment), male sex, diabetes duration (per 1-year increment), and history of hypoglycemia, with rate ratios (95% CIs) for microvascular complications of 1.14 (1.09–1.19), 1.30 (1.20–1.42), 1.03 (1.02–1.04) and 1.45 (1.25–1.69), respectively, and for macrovascular complications of 1.41 (1.34–1.48), 1.29 (1.16–1.45), 1.02 (1.01–1.02) and 1.24 (1.04–1.48), respectively. HbA_1c_ levels (per 1.0% increment) were positively associated with microvascular (1.05 [1.02–1.08]) but not macrovascular (1.00 [0.97–1.04]) complications.

**Conclusions:**

The global burden of microvascular and macrovascular complications is substantial in these patients with type 2 diabetes who are relatively early in the disease process. These findings highlight an opportunity for aggressive early risk factor modification, particularly in regions with a high prevalence of complications.

*Trial registration* ClinicalTrials.gov; NCT02322762. Registered 23 December 2014. https://clinicaltrials.gov/ct2/show/NCT02322762. ClinicalTrials.gov; NCT02226822. Registered 27 August 2014. https://clinicaltrials.gov/ct2/show/NCT02226822

**Electronic supplementary material:**

The online version of this article (10.1186/s12933-018-0787-8) contains supplementary material, which is available to authorized users.

## Background

Type 2 diabetes is associated with disabling and potentially life-threatening microvascular and macrovascular complications [1, 2]. As many as 80% of patients with type 2 diabetes develop cardiovascular complications, which account for approximately 65% of deaths in this group [3–5]. The contribution of microvascular complications to type 2 diabetes morbidity is also substantial [2, 6]. Large prospective studies have demonstrated that intensive glycemic control decreases the incidence and delays the progression of microvascular complications in patients with type 2 diabetes [7–12]. The benefits of intensive glycemic control in reducing the incidence of macrovascular complications are less clear; however, previous studies have demonstrated that better glycemic control may modestly reduce the long-term risk of some macrovascular events. Furthermore, recent cardiovascular outcome trials [13, 14] and a large observational study [15] have suggested that some glucose-lowering agents may substantially improve cardiovascular outcomes in patients with type 2 diabetes.

Previous estimates from observational studies of the prevalence of vascular complications in patients with type 2 diabetes vary greatly [16–21], and a lack of standardization in the assessment methods used renders them difficult to compare. Better understanding of the burden of vascular complications in patients with type 2 diabetes across the globe is of considerable importance, particularly in countries where no data are currently available, because this will provide critically important information for decisions on health policy, including strategies for complication prevention.

There is a paucity in particular regarding global, comparative data on baseline characteristics, treatment pathways and outcomes in patients with type 2 diabetes who are relatively early in the disease process at the time of initiating second-line glucose-lowering therapy. It is particularly important to obtain comparative data, given the plethora of second-line treatment options for patients with type 2 diabetes, compared with the first-line setting in which metformin monotherapy is well established and recommended by major treatment guidelines. To address this important knowledge gap, we initiated DISCOVER (NCT02322762, NCT02226822), a 3-year, prospective, observational study program of patients with type 2 diabetes initiating second-line therapy, conducted in 38 countries across six continents [22, 23]. Here, we report the baseline data from DISCOVER to describe the prevalence of vascular complications in patients with type 2 diabetes across multiple countries and regions, using standardized methodology.

## Methods

The methods for the DISCOVER study have been reported in detail elsewhere [22, 23] and are briefly summarized below.

### Study design

The DISCOVER program is a noninterventional, 3-year, prospective observational study program conducted across 38 countries and comprising two similar studies: DISCOVER in 37 countries (NCT02322762) and J-DISCOVER in Japan (NCT02226822). The study protocol was approved by the appropriate clinical research ethics committees in each participating country and the relevant institutional review boards at each site. The protocol complies with the Declaration of Helsinki, the International Conference on Harmonisation of Good Clinical Practice, and the local regulations for clinical research.

### Countries and regional definition

The countries included in the study were separated into regions according to World Health Organization classification [24]: Africa (Algeria and South Africa); the Americas (Argentina, Brazil, Canada, Colombia, Costa Rica, Mexico, and Panama); South-East Asia (India and Indonesia); Europe (Austria, Czech Republic, Denmark, France, Italy, Netherlands, Norway, Poland, Russia, Spain, Sweden, and Turkey); the Eastern Mediterranean region (Bahrain, Egypt, Jordan, Kuwait, Lebanon, Oman, Saudi Arabia, Tunisia, and United Arab Emirates); and the Western Pacific region (Australia, China, Japan, Malaysia, South Korea, and Taiwan).

### Site and investigator selection

National coordinating investigators of participating countries provided information on the management of patients with type 2 diabetes in their country, including types of physicians (primary care practitioners, diabetologists, endocrinologists, cardiologists, and other specialists), types of practices (primary care centers and different types of hospital), locations of practices (urban or rural), and geographical distribution within each country. Information from the national coordinating investigators and data from literature searches were reviewed to establish a list of sites that would be as representative as possible of the management of patients with type 2 diabetes in each country. Sites were then invited to participate, and those that accepted were included in the study.

### Patient enrollment

All inclusion and exclusion criteria are shown in Additional file 1: Table S1. Eligible patients with type 2 diabetes initiating a second-line glucose-lowering treatment (add-on or switching) after a first-line oral monotherapy or combination therapy were invited to participate in the study by their physician. Patients using an injectable agent (i.e. insulin or a glucagon-like peptide-1 receptor agonist) as first-line therapy were excluded because they may have had a more severe disease profile that would warrant a separate study. All participating patients provided signed informed consent.

### Data collection

Baseline data were collected at initiation of second-line therapy. In most countries, the investigators collected data using a standardized electronic case report form, and data were transferred to a central database via a web-based data capture system. In the process of data cleaning and preparation for analyses, data were checked for internal consistency and outliers (impossible values) were identified. Variables recorded at baseline included: demographic and socioeconomic characteristics, physiological parameters (blood pressure, pulse rate, weight, height, body mass index [BMI] and waist circumference), change in glucose-lowering therapy and reason(s) for change, glycated hemoglobin (HbA_1c_) level and other laboratory parameters (blood test and urine test results [22]), occurrence of major and minor hypoglycemic events, comorbidities (including microvascular and macrovascular complications), comedications, and patient-reported outcomes. Duration of diabetes was assessed as time since diagnosis. In line with the observational nature of the study, information with regard to glycemic control and other clinical variables was collected as measured in routine clinical practice at each site, according to the local standard of care. Similarly, the study protocol did not mandate the screening for or adjudication of complication occurrence.

### Vascular complications

Investigators collected information on patients’ history of complications or related procedures from medical records. Patients who had experienced either micro- or macrovascular disease prior to their diabetes diagnosis were not excluded. Diagnosis and classification of complications relied on the judgment of investigators. The following vascular complications were assessed:

- microvascular: nephropathy (presence of chronic kidney disease and/or albuminuria), retinopathy (history of retinopathy or retinal laser photocoagulation), and neuropathy (autonomic neuropathy, peripheral neuropathy, and erectile dysfunction)
- macrovascular: coronary artery disease (history of coronary artery disease, angina, myocardial infarction, percutaneous coronary intervention, and coronary artery bypass grafting), cerebrovascular disease (stroke, transient ischemic attack, carotid artery stenting, and carotid endarterectomy), peripheral artery disease (history of peripheral artery disease including revascularization procedures, diabetic foot, and amputation), heart failure, and implantable cardioverter defibrillator use.

### Statistical analysis

The minimum sample size was estimated to be 11,100 [22]. This sample size was based on the intention to have at least 200 patients comprising any given group or meeting any of the pre-specified endpoints to be analyzed, and an estimated attrition rate of 15% per year of follow-up, as described in detail elsewhere [22]. Descriptive data are presented as numbers (percentages), means (standard deviations [SD]), and medians (interquartile ranges [IQR]), as appropriate. The crude prevalence of microvascular and macrovascular complications is reported overall, and by country and region. In order to correct for geographical variations, regional and country-level prevalence estimates were also standardized for age and sex using a logistic regression model.

Factors associated with complications were assessed using a modified Poisson model with cluster-based sandwich variance estimator [25], to account for patient clustering within countries. The following variables were included in the model, based on a literature review and clinical judgment: age, sex, education level, smoking status, BMI, systolic blood pressure (SBP), HbA_1c_, total cholesterol, duration of diabetes, history of hypoglycemia (minor event in the previous month or major event in the previous year), and use of angiotensin-converting enzyme (ACE) inhibitors or angiotensin receptor blockers (ARBs), diuretics, β-blockers, statins, and acetylsalicylic acid (ASA). Data were reported in > 90% of patients for all of these variables except HbA_1c_ (not reported in 20.1% of patients) and total cholesterol (not reported in 42.3% of patients). To account for unreported data, the multiple imputation method was used for missing values. A comparison of the characteristics of patients with reported data for all variables included in the modified Poisson model, and patients with unreported data for either HbA_1c_ or total cholesterol, is shown in Additional file 1: Table S2. The following sensitivity analyses were conducted: (1) an analysis including only patients with complete data; and (2) an analysis with additional variables for site specialty and patient-reported ethnicity (ethnicity and specialty information was not collected in Canada, and specialty was not collected in France; these two countries were thus excluded from this analysis). Rate ratios for the associations between complication prevalence and SBP, total cholesterol levels, and comedication use were not reported owing to reverse-causality (we hypothesized that patients with diabetes complications often have high SBP and cholesterol levels and receive comedications, rather than these factors causing vascular complications), although these factors were adjusted for in the model.

Imputation was carried out using IVEware (University of Michigan, MI, USA). All other statistical analyses were carried out using the SAS statistical software system (SAS Institute, Inc., Cary, NC, USA).

## Results

### Baseline patient characteristics

The overall population included 15,992 patients. Baseline characteristics are presented in Table 1. In total, 54.2% of patients were male; 49.7% were Asian, and 25.6% were Caucasian. The mean age was 57.2 years (SD, 12.0 years), ranging from 53.1 years in South-East Asia to 61.9 years in Europe. The median duration of diabetes was 4.1 years (IQR across countries: 1.9–7.9 years), varying across regions from 3.4 years in South-East Asia and the Western Pacific region to 5.7 years in Africa. The median HbA_1c_ level at baseline was 8.0% (IQR across countries: 7.2–9.1%) and varied across regions from 7.6% in the Western Pacific to 8.3% in South-East Asia and the Eastern Mediterranean region. The mean BMI was 29.1 kg/m^2^ (SD, 5.9 kg/m^2^) and was lowest in the Western Pacific region (26.1 kg/m^2^) and highest in Europe (31.9 kg/m^2^).

**Table 1:** Patient demographics and baseline characteristics, overall and according to region

	Total (N = 15,992)	Africa (n = 812)	Americas (n = 2002)	South-East Asia (n = 3360)	Europe (n = 3479)	Eastern Mediterranean (n = 2182)	Western Pacific (n = 4157)
Proportion of overall population (%)	100.0	5.1	12.5	21.0	21.8	13.6	26.0
Sex, male, n (%)	8664 (54.2)	306 (37.7)	963 (48.1)	1852 (55.1)	1856 (53.4)	1278 (58.6)	2409 (58.0)
Age, years, mean (SD)	57.2 (12.0)	54.9 (11.2)	58.3 (11.8)	53.1 (11.3)	61.9 (10.9)	53.8 (10.8)	58.5 (12.6)
Self-reported ethnicity, n (%)							
Caucasian	3917 (25.6)	105 (12.9)	480 (29.4)	1 (0.0)	3020 (94.8)	165 (7.6)	146 (3.5)
Black	310 (2.0)	235 (29.0)	61 (3.7)	0 (0.0)	13 (0.4)	0 (0.0)	1 (0.0)
Mixed	213 (1.4)	91 (11.2)	115 (7.0)	0 (0.0)	4 (0.1)	0 (0.0)	3 (0.1)
Asian	7610 (49.7)	177 (21.8)	9 (0.6)	3339 (99.5)	20 (0.6)	72 (3.3)	3993 (96.1)
Hispanic	942 (6.2)	1 (0.1)	928 (56.8)	0 (0.0)	11 (0.3)	0 (0.0)	2 (0.0)
Arabic	2151 (14.0)	200 (24.7)	4 (0.2)	2 (0.1)	12 (0.4)	1933 (88.9)	0 (0.0)
Other	174 (1.1)	2 (0.2)	36 (2.2)	15 (0.4)	104 (3.3)	5 (0.2)	12 (0.3)
If Asian							
Chinese	1604 (21.1)	0 (0.0)	1 (11.1)	52 (1.6)	2 (10.5)	0 (0.0)	1549 (38.8)
South Asian	2602 (34.2)	60 (33.9)	2 (22.2)	2406 (72.1)	9 (47.4)	46 (63.9)	79 (2.0)
East Asian	433 (5.7)	22 (12.4)	1 (11.1)	48 (1.4)	5 (26.3)	16 (22.2)	341 (8.5)
Other Asian	2971 (39.0)	95 (53.7)	5 (55.6)	833 (24.9)	3 (15.8)	10 (13.9)	2025 (50.7)
Time in formal education, n (%)							
No formal education	471 (3.2)	57 (7.3)	50 (3.2)	26 (0.8)	78 (2.5)	158 (7.7)	102 (2.7)
Primary (1–6 years)	2295 (15.8)	183 (23.3)	442 (28.7)	343 (10.4)	588 (19.1)	360 (17.6)	379 (10.0)
Secondary (7–13 years)	7190 (49.4)	420 (53.5)	587 (38.1)	1431 (43.2)	1781 (58.0)	767 (37.5)	2204 (58.0)
Higher (> 13 years)	4599 (31.6)	125 (15.9)	463 (30.0)	1514 (45.7)	626 (20.4)	759 (37.1)	1112 (29.3)
Diabetes duration since diagnosis, years							
Mean (SD)	5.6 (5.3)	6.9 (5.7)	6.2 (6.1)	4.6 (4.1)	6.6 (5.4)	5.8 (5.1)	5.1 (5.4)
Median (IQR)	4.1 (1.9–7.9)	5.7 (2.9–9.3)	4.4 (1.9–8.7)	3.4 (2.0–6.1)	5.4 (2.7–9.1)	4.2 (2.1–8.0)	3.4 (1.0–7.6)
HbA_1c_ (%)							
Mean (SD)	8.3 (1.7)	8.6 (1.9)	8.5 (1.9)	8.6 (1.7)	8.1 (1.6)	8.7 (1.6)	8.1 (1.7)
Median (IQR)	8.0 (7.2–9.1)	8.0 (7.4–9.4)	8.0 (7.2–9.4)	8.3 (7.5–9.6)	7.8 (7.2–8.7)	8.3 (7.6–9.4)	7.6 (7.0–8.7)
BMI, kg/m^2^, mean (SD)	29.1 (5.9)	30.6 (6.2)	30.6 (6.1)	27.3 (4.5)	31.9 (6.1)	31.1 (5.7)	26.1 (5.0)
Tobacco smoking, n (%)							
Nonsmoker	10,831 (69.4)	633 (78.9)	1299 (66.1)	3066 (91.7)	2001 (59.9)	1579 (74.0)	2253 (56.1)
Ex-smoker	2537 (16.3)	93 (11.6)	460 (23.4)	128 (3.8)	791 (23.7)	189 (8.9)	876 (21.8)
Current smoker	2232 (14.3)	76 (9.5)	205 (10.4)	151 (4.5)	546 (16.4)	366 (17.2)	888 (22.1)
SBP, mmHg, mean (SD)	132.3 (16.5)	134.2 (18.6)	131.2 (17.7)	128.8 (15.2)	136.4 (16.6)	133.3 (15.7)	131.6 (16.0)
DBP, mmHg, mean (SD)	79.9 (10.0)	80.1 (10.5)	80.6 (10.6)	79.9 (8.4)	81.1 (9.6)	79.8 (9.7)	78.6 (10.9)
TC, mg/dl, mean (SD)	187.0 (47.1)	179.1 (41.1)	182.1 (46.1)	179.8 (48.7)	190.6 (49.9)	189.6 (47.8)	189.6 (43.7)
History of hypoglycemia^a^, n (%)	700 (4.6)	50 (6.4)	60 (3.8)	96 (2.9)	141 (4.2)	155 (7.8)	198 (4.9)
Comedication, n (%)							
ACEi or ARB	5862 (36.7)	315 (38.8)	827 (41.3)	990 (29.5)	1785 (51.3)	715 (32.8)	1230 (29.6)
Diuretic	1867 (11.7)	223 (27.5)	250 (12.5)	203 (6.0)	780 (22.4)	197 (9.0)	214 (5.1)
β-blocker	2158 (13.5)	87 (10.7)	277 (13.8)	257 (7.6)	978 (28.1)	277 (12.7)	282 (6.8)
Statin	6710 (42.0)	360 (44.3)	826 (41.3)	1497 (44.6)	1534 (44.1)	997 (45.7)	1496 (36.0)
ASA	2562 (16.0)	214 (26.4)	374 (18.7)	278 (8.3)	776 (22.3)	485 (22.2)	435 (10.5)
First-line therapy, n (%)							
MET monotherapy	9076 (56.8)	679 (83.6)	1545 (77.2)	1505 (44.8)	2517 (72.4)	1063 (48.7)	1767 (42.5)
SU monotherapy	1230 (7.7)	27 (3.3)	137 (6.8)	186 (5.5)	269 (7.7)	288 (13.2)	323 (7.8)
DPP-4i monotherapy	1194 (7.5)	1 (0.1)	40 (2.0)	43 (1.3)	53 (1.5)	19 (0.9)	1038 (25.0)
Other monotherapy	631 (3.9)	2 (0.2)	17 (0.8)	36 (1.1)	59 (1.7)	13 (0.6)	504 (12.1)
MET + SU	2300 (14.4)	73 (9.0)	135 (6.7)	1045 (31.1)	287 (8.3)	518 (23.8)	242 (5.8)
MET + DPP-4i	497 (3.1)	1 (0.1)	92 (4.6)	123 (3.7)	94 (2.7)	130 (6.0)	57 (1.4)
MET + other (dual therapy)	266 (1.7)	18 (2.2)	3 (0.1)	61 (1.8)	56 (1.6)	16 (0.7)	112 (2.7)
Other combinations	794 (5.0)	11 (1.4)	33 (1.6)	361 (10.7)	141 (4.1)	134 (6.1)	114 (2.7)

Percentages calculated for all patients with data available; unreported data are excluded

*ACEi* angiotensin-converting-enzyme inhibitor, *ARB* angiotensin receptor blocker, *ASA* acetylsalicylic acid, *BMI* body mass index, *DBP* diastolic blood pressure, *DPP*-*4i* dipeptidyl peptidase-4 inhibitor, *HbA*_*1c*_ glycated hemoglobin, *IQR* interquartile range, *MET* metformin, *SBP* systolic blood pressure, *SD* standard deviation, *SU* sulfonylurea, *TC* total cholesterol

^a^Minor hypoglycemic event in the previous month or major hypoglycemic event in the previous year

The most common first-line therapy was metformin monotherapy, both overall (56.8%) and across all regions (range 42.5–83.6%). The second most commonly prescribed first-line therapy was sulfonylurea monotherapy in the Americas (6.8%), dipeptidyl peptidase-4 inhibitor monotherapy in the Western Pacific region (25.0%), and combinations of metformin and a sulfonylurea in other regions (range 8.3–31.1%).

### Prevalence of microvascular complications

**Fig. 1:**
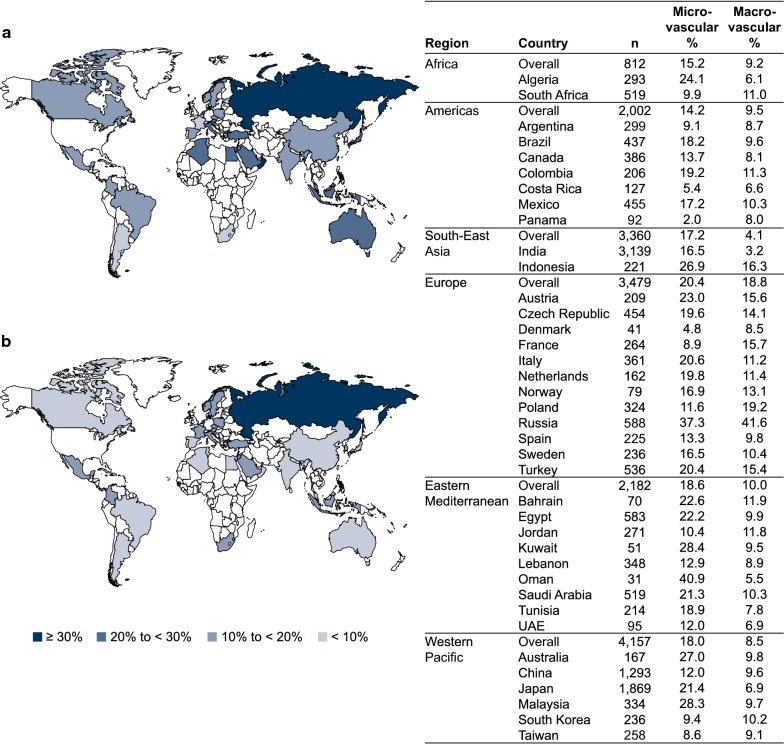
Age- and sex-standardized prevalence of **a** microvascular and **b** macrovascular complications, according to region and country. Percentages calculated for all patients with data available; unreported data are excluded. *UAE* United Arab Emirates

The crude prevalence of microvascular complications was 18.8% overall; it was greatest in Europe (23.5%) and lowest in Africa (14.5%; Table 2). Crude country-level prevalence estimates are shown in Additional file 1: Table S3. The crude prevalence was 7.7% for peripheral neuropathy, 5.0% for chronic kidney disease, 4.3% for albuminuria, 3.9% for retinopathy, 2.7% for erectile dysfunction, 1.0% for autonomic neuropathy and 0.6% for retinal laser photocoagulation (Table 2). Peripheral neuropathy was the most prevalent microvascular disease diagnosis in all regions except the Western Pacific (the most prevalent microvascular disease in the Western Pacific was chronic kidney disease). After standardization for age and sex, the prevalence of microvascular complications was 17.9% (95% confidence interval [CI] 17.3–18.6%) overall; it remained highest in Europe (20.4% [95% CI 19.0–22.0%]) and was lowest in the Americas (14.2% [95% CI 12.7–16.0%]), ranging across countries from 2.0 to 40.9% (Fig. 1a). Within Europe, the age- and sex-standardized prevalence of microvascular complications was 37.3% in Russia and ranged from 4.8 to 23.0% in the other countries in the region. When excluding Russia from the analysis, the prevalence in Europe was 16.9%.

**Table 2:** Crude prevalence of microvascular or macrovascular diseases and related procedures at baseline, overall and according to region

	Total (N = 15,992)	Africa (n = 812)	Americas (n = 2002)	South-East Asia (n = 3360)	Europe (n = 3479)	Eastern Mediterranean (n = 2182)	Western Pacific (n = 4157)
Any microvascular disease, n (%)	3005 (18.8)	118 (14.5)	302 (15.1)	556 (16.5)	812 (23.5)	399 (18.3)	818 (19.7)
CKD	794 (5.0)	20 (2.5)	90 (4.5)	23 (0.7)	257 (7.4)	31 (1.4)	373 (9.0)
Albuminuria	605 (4.3)	21 (2.6)	40 (2.0)	188 (5.6)	195 (5.6)	91 (4.2)	70 (3.1)
Retinopathy	624 (3.9)	22 (2.7)	68 (3.4)	30 (0.9)	198 (5.7)	69 (3.2)	237 (5.7)
Retinal laser photocoagulation	98 (0.6)	4 (0.5)	17 (0.8)	1 (0.0)	14 (0.4)	9 (0.4)	53 (1.3)
Autonomic neuropathy	155 (1.0)	1 (0.1)	9 (0.4)	40 (1.2)	28 (0.8)	25 (1.1)	52 (1.3)
Peripheral neuropathy	1237 (7.7)	47 (5.8)	114 (5.7)	324 (9.6)	324 (9.4)	181 (8.3)	247 (5.9)
Erectile dysfunction	426 (2.7)	38 (4.7)	65 (3.2)	28 (0.8)	115 (3.3)	102 (4.7)	78 (1.9)
Any macrovascular disease, n (%)	2027 (12.7)	74 (9.1)	232 (11.6)	135 (4.0)	915 (26.7)	218 (10.0)	453 (10.9)
Heart failure	527 (3.3)	7 (0.9)	54 (2.7)	18 (0.5)	368 (10.7)	23 (1.1)	57 (1.4)
CAD	1310 (8.2)	59 (7.3)	151 (7.5)	91 (2.7)	622 (18.1)	172 (7.9)	215 (5.2)
Angina	473 (3.0)	19 (2.3)	53 (2.6)	10 (0.3)	262 (7.6)	40 (1.8)	89 (2.1)
Myocardial infarction	445 (2.8)	39 (4.8)	80 (4.0)	18 (0.5)	228 (6.6)	31 (1.4)	49 (1.2)
PCI	424 (2.7)	10 (1.2)	62 (3.1)	19 (0.6)	184 (5.4)	70 (3.2)	79 (1.9)
CABG	140 (0.9)	10 (1.2)	17 (0.8)	13 (0.4)	71 (2.1)	15 (0.7)	14 (0.3)
Stroke	352 (2.2)	4 (0.5)	35 (1.7)	20 (0.6)	114 (3.3)	29 (1.3)	150 (3.6)
Transient ischemic attack	107 (0.7)	3 (0.4)	14 (0.7)	1 (0.0)	36 (1.0)	17 (0.8)	36 (0.9)
Carotid artery stent	16 (0.1)	0 (0.0)	1 (0.0)	0 (0.0)	7 (0.2)	4 (0.2)	4 (0.1)
Carotid endarterectomy	16 (0.1)	0 (0.0)	0 (0.0)	0 (0.0)	12 (0.3)	1 (0.0)	3 (0.1)
PAD	197 (1.2)	4 (0.5)	15 (0.7)	2 (0.1)	108 (3.1)	10 (0.5)	58 (1.4)
Diabetic foot	87 (0.5)	4 (0.5)	20 (1.0)	15 (0.4)	32 (0.9)	7 (0.3)	9 (0.2)
Amputation	32 (0.2)	6 (0.7)	12 (0.6)	1 (0.0)	9 (0.3)	3 (0.1)	1 (0.0)
Defibrillator use	8 (0.1)	0 (0.0)	0 (0.0)	0 (0.0)	7 (0.2)	0 (0.0)	1 (0.0)

Percentages calculated for all patients with data available; unreported data are excluded

*CABG* coronary artery bypass grafting, *CAD* coronary artery disease, *CKD* chronic kidney disease, *PAD* peripheral artery disease, *PCI* percutaneous coronary intervention

### Prevalence of macrovascular complications

The crude prevalence of macrovascular complications was 12.7% overall and was greatest in Europe (26.7%) and lowest in South-East Asia (4.0%; Table 2). Crude country-level prevalence estimates are shown in Additional file 1: Table S3. The crude prevalence was 8.2% for coronary artery disease, 3.3% for heart failure, 2.2% for stroke, 1.2% for peripheral artery disease, 0.7% for transient ischemic attack, and 0.1% each for carotid artery stent, carotid endarterectomy, and defibrillator use (Table 2). After standardization for age and sex, the prevalence of macrovascular complications was 9.2% (95% CI 8.7–9.7%) overall; it remained greatest in Europe (18.8% [95% CI 17.4–20.3%]) and lowest in South-East Asia (4.1% [95% CI 3.5–4.9%]), ranging across countries from 3.2 to 41.6% (Fig. 1b). When excluding Russia from the analysis, the age- and sex-standardized prevalence of macrovascular complications in Europe was 13.7% and remained the highest across the regions.

The most prevalent macrovascular complication in all regions was coronary artery disease (2.7–18.1% across regions; 8.2% overall), with myocardial infarction most reported in Africa, the Americas, and Europe (4.0–6.6%), and percutaneous coronary intervention most reported in South-East Asia, the Eastern Mediterranean region, and the Western Pacific (0.6–3.2%). The most frequently reported macrovascular complications, after coronary artery disease and its components, were heart failure in Africa, the Americas, and Europe (0.9–10.7%), and stroke in South-East Asia, the Eastern Mediterranean region, and the Western Pacific (0.6–3.6%).

### Multivariable analysis

**Fig. 2:**
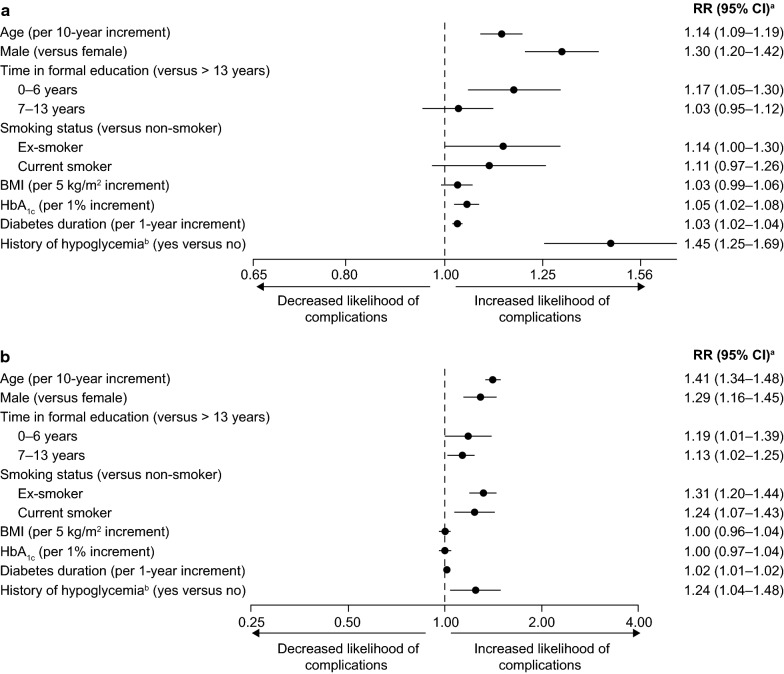
Multivariable analysis of factors associated with **a** microvascular and **b** macrovascular complications. ^a^RRs adjusted for all variables in the figure with the addition of SBP, total cholesterol levels and comedication use, using a modified Poisson model with cluster-based sandwich variance estimator as described in “Methods”. RRs for the associations between complication prevalence and SBP, total cholesterol levels, and comedication use are not reported due to reverse-causality. ^b^Minor hypoglycemic event in the previous month or major hypoglycemic event in the previous year. *BMI* body mass index, *CI* confidence interval, *HbA*_*1c*_ glycated hemoglobin, *RR* rate ratio

Similar factors were found to have a statistically significant positive association with the prevalence of both microvascular and macrovascular complications (Fig. 2). These included age (per 10-year increment; microvascular—rate ratio: 1.14 [95% CI 1.09–1.19]; macrovascular—rate ratio: 1.41 [95% CI 1.34–1.48]), male sex (1.30 [1.20–1.42] and 1.29 [1.16–1.45]), having a low level of education (0–6 years in formal education relative to > 13 years; 1.17 [1.05–1.30] and 1.19 [1.01–1.39]), duration of diabetes since diagnosis (per 1-year increment; 1.03 [1.02–1.04] and 1.02 [1.01–1.02]), and having a history of any hypoglycemic event (minor event in the previous month or major event in the previous year; 1.45 [1.25–1.69 and 1.24 [1.04–1.48]). Increased mean baseline HbA_1c_ levels (per 1.0% increment) were positively associated with microvascular (rate ratio: 1.05 [95% CI 1.02–1.08]) but not macrovascular (rate ratio: 1.00 [95% CI 0.97–1.04]) complications. Being either a former or current smoker was positively associated with macrovascular complications (1.31 [1.20–1.40] and 1.24 [1.07–1.43]) relative to being a non-smoker.

Results from the sensitivity analyses including only patients with complete data (Additional file 1: Figure S1) and with additional variables for site specialty and patient-reported ethnicity (Additional file 1: Figure S2) were similar to those from the main analysis. Specialty and ethnicity were not associated with microvascular or macrovascular complications, with the exception of lower rates of macrovascular complications with self-reported black versus Caucasian ethnicity and higher rates of macrovascular complications with self-reported East Asian versus Caucasian ethnicity.

## Discussion

This large, prospective study of close to 16,000 patients with type 2 diabetes initiating second-line glucose-lowering therapy is being conducted in 38 countries across six continents. The burden of both microvascular and macrovascular complications at second-line therapy initiation, assessed using a standardized methodology, was found to be substantial and varied markedly between regions and countries. This study is one of the first to offer a truly global view of the prevalence of vascular complications among patients with type 2 diabetes who are at relatively early stages of their disease. DISCOVER also includes many lower- and upper-middle-income countries, for which no data on the prevalence of diabetes complications were previously available.

When standardized for age and sex, the highest prevalence of microvascular and macrovascular complications was found in Europe, where patients also had the highest mean BMI and blood pressure, which are important cardiovascular risk factors [26]. These findings could also be explained by rates of screening for complications being higher in Europe than in other regions, or by a greater proportion of patients seen in specialty settings. Across the European countries, Russia had the highest prevalence of microvascular and macrovascular complications. These observations are supported by Russian state diabetes registry data, which show a high prevalence of recorded vascular disease in patients with type 2 diabetes, including 18.6% for diabetic neuropathy and 13.0% for diabetic retinopathy [27]. When excluding Russia from our analysis, the prevalence in Europe of macrovascular complications, but not that of microvascular complications, remained the highest across all regions.

Our multivariable analysis of factors potentially associated with complication prevalence identified positive correlations between microvascular and macrovascular complications and age, male sex, low level of education, diabetes duration, and history of hypoglycemia. Other research has shown that greater and more prolonged exposure to hyperglycemia, as would occur in patients with a long duration of uncontrolled type 2 diabetes, increases the risk of both microvascular and macrovascular complications [28]. Of note, a significant association was observed in the current analysis between HbA_1c_ level and microvascular, but not macrovascular, complications. This is consistent with previous findings that intensive glycemic control decreases the incidence of microvascular complications, with much less certainty in terms of its effects on macrovascular events [7–12, 29].

Large, international, observational studies that have reported the prevalence of vascular complications in patients with type 2 diabetes are rare. The A_1_chieve study was a global, prospective, observational study of more than 66,000 patients with type 2 diabetes from 28 countries in Asia, Africa, Europe, and South America, and reported a prevalence of microvascular complications at baseline of 53.5%, and of macrovascular complications of 27.2% [21]. However, the patients included in that study were initiating insulin analogs, typically administered after failure to achieve target HbA_1c_ levels with other glucose-lowering drugs. Consequently, the patients in A_1_chieve had a much longer duration of diabetes than those in DISCOVER (mean 8.0 vs 5.6 years) and a higher mean baseline HbA_1c_ level (9.5% vs 8.4%).

The IMPROVE study, conducted in eight countries and involving more than 50,000 patients receiving insulin therapy, also reported a high prevalence of both microvascular and macrovascular complications (45.0% and 28.0%, respectively) [30]. Again, the mean duration of diabetes (6.9 years) and mean HbA_1c_ levels (9.4%) were higher in IMPROVE than in the DISCOVER study. The International Diabetes Management Practice Study (IDMPS) was conducted in 18 developing countries across Asia, Eastern Europe, and Latin America. The prevalence estimates reported for microvascular and macrovascular complications were again high at 55.3% and 26.1%, respectively [31]. In this survey of a general population of patients with type 2 diabetes, the mean diabetes duration was 8.4 years, and 31% of patients were treated with insulin, either on its own or in combination with oral agents.

The differences in patient characteristics between A_1_chieve, IMPROVE, IDMPS, and DISCOVER are likely to explain the differences in the observed rates of vascular complications. All three of the previous studies included patients with longer-term disease than in DISCOVER. Our study is therefore the first global observational study of its kind, evaluating a population of patients with a much shorter disease duration.

Our findings highlight a key opportunity for improved monitoring of complications, and the importance of early and aggressive risk factor modification. This is in line with current practice guidelines; for example, the European Society of Cardiology guidelines for cardiovascular disease prevention consider patients with diabetes to be at very high cardiovascular risk, regardless of other risk factors, and therefore recommend statin treatment in addition to intensive blood pressure management for all patients [32]. The American Heart Association and American Diabetes Association similarly recommend intensive management of cardiovascular risk factors in patients with diabetes [33]. As outlined in the American Diabetes Association/European Association for the Study of Diabetes joint position statement, it is also important to consider classes of glucose-lowering medications with positive impacts on cardiovascular risk factors, especially in patients with established cardiovascular disease [34]. In a recent, large cohort study, patients with type 2 diabetes who had levels of HbA_1c_, cholesterol, serum albumin and blood pressure all within target ranges, and who did not smoke, were at similar risk of myocardial infarction or stroke as age-, sex- and country-matched controls [35]. Risk scores can help clinicians identify patients with type 2 diabetes who are at low or high risk of vascular disease [36, 37].

The main strengths of the DISCOVER study include its prospective design, the large number of patients enrolled, and the participation of lower- and upper-middle-income countries where patients have rarely, or never, been studied previously. The use of a standardized electronic case report form is another key strength of the study, allowing valid comparisons of results within and across countries and regions worldwide. In addition, sites were selected in a way intended to ensure that the enrolled patient population is as diverse and representative as possible of the general population of patients with type 2 diabetes.

Our findings should, nevertheless, be interpreted in the context of several potential limitations. The ascertainment and diagnosis of diabetes-related vascular complications were based on the judgment of individual physicians, and we could not determine whether the complications occurred before or after the diagnosis of type 2 diabetes. In some countries, rural sites do not have any established infrastructure for data collection, and primary care practitioners may be insufficiently trained, or not permitted, to conduct observational research. In these countries, where rural sites and primary care settings are necessarily under-represented, the quality of healthcare is likely to be overestimated.

The observational nature of the DISCOVER study means that the study protocol does not mandate screening for complications, and that the presence and severity of complications are not adjudicated. The reported prevalence estimates may therefore be underestimates, because physicians may not be aware of complications in some patients. In many lower- and upper-middle-income countries participating in DISCOVER, fundus cameras used for retinopathy screening may be inaccessible to many patients, particularly in rural areas [38].

In the present analysis, we considered erectile dysfunction to be a microvascular complication, and diabetic foot to be a macrovascular complication. However, no assessment of the underlying pathophysiology of these conditions in individual patients was made. Finally, imputation was required during the multivariable analysis to account for some unreported data, in particular for HbA_1c_ and total cholesterol. This is consistent with the noninterventional nature of the DISCOVER study, whereby laboratory values and other clinical variables are measured according to standard clinical practice at each site. In several countries, measurement of biomarkers including HbA_1c_ and total cholesterol may not be covered by health insurance, and may therefore not be routinely measured for all patients.

## Conclusions

The prevalence of microvascular and macrovascular complications in patients with type 2 diabetes at second-line therapy initiation, assessed using a standardized methodology, varied markedly across regions and countries worldwide, and highlighted significant opportunities for better risk factor modification and prevention. Across all regions, Europe had the highest prevalence of both microvascular complications and macrovascular complications, the former being driven by high rates in Russia. Over the coming years, the DISCOVER study program will add to our understanding by providing follow-up longitudinal data on the incidence of complications following initiation of second- and later-line therapies.

## Additional file

**Additional file 1: Table S1.** Inclusion and exclusion criteria. DDP-4 dipeptidyl peptidase-4. ^a^≥ 20 years in Japan. ^b^In Japan, only patients using an oral monotherapy as first-line treatment were included. **Table S2.** Comparisons between patients for whom either HbA_1c_ or total cholesterol data are unreported, and those with complete HbA_1c_ and total cholesterol data. *ACEi* angiotensin-converting-enzyme inhibitor, *ARB* angiotensin receptor blocker, *ASA* acetylsalicylic acid, *BMI* body mass index, *HbA*_*1c*_ glycated hemoglobin, *SBP* systolic blood pressure, *SD* standard deviation, *TC* total cholesterol. ^a^Patients with reported data for all variables included in the hierarchical logistic model. ^b^P values calculated for continuous variables using Student’s *t*-test, and for categorical variables using the χ^2^ or Fisher’s exact test, as appropriate. ^c^Minor hypoglycemic event in the previous month or major hypoglycemic event in the previous year. **Table S3.** Number and proportion of patients with microvascular and macrovascular complications according to country (unadjusted). Percentages were calculated for all patients with data available; unreported data were excluded. *UAE* United Arab Emirates. **Figure S1.** Sensitivity analysis including only patients with complete data to assess factors associated with (A) microvascular and (B) macrovascular complications. ^a^RRs adjusted for all variables in the figure with the addition of SBP, total, cholesterol levels and comedication use, using a modified Poisson model with cluster-based sandwich variance estimator as described in “Methods”. RRs for the associations between complication prevalence and SBP, total cholesterol levels, and comedication use are not reported due to reverse-causality. ^b^Minor hypoglycemic event in the previous month or major hypoglycemic event in the previous year. *BMI* body mass index, *CI* confidence interval, *HbA*_*1c*_ glycated hemoglobin, *RR* rate ratio. **Figure S2.** Sensitivity analysis with additional variables for site specialty and patient-reported ethnicity to assess factors associated with (A) microvascular and (B) macrovascular complications. ^a^RRs adjusted for all variables in the figure with the addition of SBP, total, cholesterol levels and comedication use, using a modified Poisson model with cluster-based sandwich variance estimator as described in “Methods”. RRs for the associations between complication prevalence and SBP, total cholesterol levels, and comedication use are not reported due to reverse-causality. ^b^Minor hypoglycemic event in the previous month or major hypoglycemic event in the previous year. *BMI* body mass index, *CI *confidence interval, *HbA*_*1c*_ glycated hemoglobin, *RR* rate ratio.

## Abbreviations

ACE: angiotensin-converting enzyme
ARB: angiotensin receptor blocker
ASA: acetylsalicylic acid
BMI: body mass index
DBP: diastolic blood pressure
DPP-4i: dipeptidyl peptidase-4 inhibitor
HbA_1c_: glycated hemoglobin
IQR: interquartile range
MET: metformin
SBP: systolic blood pressure
SD: standard deviation
SU: sulfonylurea
TC: total cholesterol
