# Supplementary material for: Vascular complications in patients with type 2 diabetes: prevalence and associated factors in 38 countries (the DISCOVER study program)
Source: Cardiovasc Diabetol. 2018 Nov 28;17:150. doi: 10.1186/s12933-018-0787-8 (PMC6260731; doi:10.1186/s12933-018-0787-8)
Supplement: Supplementary file 1 — Additional file 1: Table S1. Inclusion and exclusion criteria. DDP-4 dipeptidyl peptidase-4. a≥ 20 years in Japan. bIn Japan, only patients using an oral monotherapy as first-line treatment were included. Table S2. Comparisons between patients for whom either HbA1c or total cholesterol data are unreported, and those with complete HbA1c and total cholesterol data. ACEi angiotensin-converting-enzyme inhibitor, ARB angiotensin receptor blocker, ASA acetylsalicylic acid, BMI body mass index, HbA1c glycated hemoglobin, SBP systolic blood pressure, SD standard deviation, TC total cholesterol. aPatients with reported data for all variables included in the hierarchical logistic model. bP values calculated for continuous variables using Student’s t-test, and for categorical variables using the χ2 or Fisher’s exact test, as appropriate. cMinor hypoglycemic event in the previous month or major hypoglycemic event in the previous year. Table S3. Number and proportion of patients with microvascular and macrovascular complications according to country (unadjusted). Percentages were calculated for all patients with data available; unreported data were excluded. UAE United Arab Emirates. Figure S1. Sensitivity analysis including only patients with complete data to assess factors associated with (A) microvascular and (B) macrovascular complications. aRRs adjusted for all variables in the figure with the addition of SBP, total, cholesterol levels and comedication use, using a modified Poisson model with cluster-based sandwich variance estimator as described in “Methods”. RRs for the associations between complication prevalence and SBP, total cholesterol levels, and comedication use are not reported due to reverse-causality. bMinor hypoglycemic event in the previous month or major hypoglycemic event in the previous year. BMI body mass index, CI confidence interval, HbA1c glycated hemoglobin, RR rate ratio. Figure S2. Sensitivity analysis with additional variables for site specialty and [file 12933_2018_787_MOESM1_ESM.docx]

# ONLINE-ONLY ADDITIONAL MATERIAL

**Additional Tables**

**Table S1 Inclusion and exclusion criteria**

| Inclusion criteria |
| --- |
| - Diagnosis of type 2 diabetes - Age ≥ 18 years^a^ - Initiating a second-line therapy (add-on or switching) after a first-line oral treatment with monotherapy, dual therapy, or triple therapy^b^ - Provision of written informed consent |
| Exclusion criteria |
| - Type 1 diabetes - Pregnancy - Initiation of dual therapy after having previously received two different lines of monotherapy (e.g. initiation of a combination of a sulfonylurea and a DPP-4 inhibitor after successive metformin monotherapy and sulfonylurea monotherapy) - Current treatment with chemotherapy or oral or intravenous steroids - Undergoing dialysis or received a renal transplant - First-line treatment with insulin or another injectable agent - First-line treatment with herbal remedies or natural medicines alone - Participation in an interventional trial - Condition or circumstance that, in the opinion of the investigator, could significantly compromise the 3-year follow-up (e.g. life-threatening comorbidities, tourist, non-native language speaker or lack of understanding of the local language in countries where interpreter services are not reliably available, psychiatric disturbances, dementia, alcohol or drug abuse) - Unwilling to sign the informed consent form |

^a^ ≥ 20 years in Japan. ^b^In Japan, only patients using an oral monotherapy as first-line treatment were included.

*DDP-4* dipeptidyl peptidase-4

**Table S2 Comparisons between patients for whom either HbA_1c_ or total cholesterol data are unreported, and those with complete HbA_1c_ and total cholesterol data**

|  | **Patients with either HbA_1c_ or TC data unreported**  **(n = 7,358)** | **Patients with complete data^a^**  **(n = 6,656)** | **P value^b^** |
| --- | --- | --- | --- |
| Any microvascular disease, n (%) | 992 (13.5) | 1,615 (24.3) | < 0.001 |
| Unreported | 14 | 1 |  |
| Any macrovascular disease, n (%) | 895 (12.2) | 856 (12.9) | 0.228 |
| Unreported | 25 | 10 |  |
| Sex, n (%) |  |  | < 0.001 |
| Male | 3,653 (49.7) | 3,842 (57.7) |  |
| Female | 3,703 (50.3) | 2,814 (42.3) |  |
| Unreported | 2 | 0 |  |
| Age, years, mean (SD) | 57.0 (12.1) | 57.2 (12.0) | 0.460 |
| Unreported | 0 | 0 |  |
| Time in formal education, n (%) |  |  | 0.039 |
| No formal education | 245 (3.6) | 193 (2.9) |  |
| Primary (1–6 years) | 1,104 (16.2) | 1,011 (15.2) |  |
| Secondary (7–13 years) | 3,333 (48.9) | 3,310 (49.7) |  |
| Higher (> 13 years) | 2,138 (31.3) | 2,142 (32.2) |  |
| Unreported | 538 | 0 |  |
| Diabetes duration, years, mean (SD) | 5.7 (5.4) | 5.6 (5.1) | 0.064 |
| Unreported | 176 | 0 |  |
| HbA_1c_, %, mean (SD) | 8.3 (1.6) | 8.4 (1.7) | 0.173 |
| Unreported | 3,207 | 0 |  |
| BMI, kg/m^2^, mean (SD) | 28.8 (5.9) | 29.1 (5.8) | 0.004 |
| Unreported | 754 | 0 |  |
| Tobacco smoking, n (%) |  |  | < 0.001 |
| Nonsmoker | 5,346 (74.5) | 4,390 (66.0) |  |
| Ex-smoker | 981 (13.7) | 1,180 (17.7) |  |
| Current smoker | 849 (11.8) | 1,086 (16.3) |  |
| Unreported | 182 | 0 |  |
| SBP, mmHg, mean (SD) | 131.7 (16.2) | 132.6 (16.8) | < 0.001 |
| Unreported | 435 | 0 |  |
| Total cholesterol, mean (SD) | 187.7 (49.4) | 187.1 (46.7) | 0.759 |
| Unreported | 6,771 | 0 |  |
| History of hypoglycemia^c^ | 327 (4.7) | 317 (4.8) | 0.834 |
| Unreported | 381 | 0 |  |
| Comedication, n (%) |  |  |  |
| ACEi or ARB | 2,406 (32.7) | 2,705 (40.6) | < 0.001 |
| Diuretic | 862 (11.7) | 798 (12.0) | 0.616 |
| β-blocker | 932 (12.7) | 947 (14.2) | 0.006 |
| Statin | 2505 (34.0) | 3311 (49.7) | < 0.001 |
| ASA | 960 (13.0) | 1,292 (19.4) | < 0.001 |
| Unreported | 0 | 0 |  |

*ACEi* angiotensin-converting enzyme inhibitor, *ARB* angiotensin receptor blocker, *ASA* acetylsalicylic acid, *BMI* body mass index, *HbA_1c_* glycated hemoglobin, *SBP* systolic blood pressure, *SD* standard deviation, *TC* total cholesterol

^a^ Patients with reported data for all variables included in the hierarchical logistic model

^b^ P values calculated for continuous variables using Student’s *t*-test, and for categorical variables using the χ^2^ or Fisher’s exact test, as appropriate

^c^ Minor hypoglycemic event in the previous month or major hypoglycemic event in the previous year

**Table S3 Number and proportion of patients with microvascular and macrovascular complications according to country (unadjusted)**

| **Region** | **Country** | **N** | **Microvascular, n (%)** | **Macrovascular, n (%)** |
| --- | --- | --- | --- | --- |
| Africa | Algeria | 293 | 70 (23.9) | 19 (6.5) |
|  | South Africa | 519 | 48 (9.2) | 55 (10.6) |
| Americas | Argentina | 299 | 30 (10.0) | 35 (11.7) |
|  | Brazil | 437 | 84 (19.2) | 52 (11.9) |
|  | Canada | 386 | 57 (14.8) | 42 (10.9) |
|  | Colombia | 206 | 44 (21.4) | 32 (15.5) |
|  | Costa Rica | 127 | 7 (5.5) | 10 (7.9) |
|  | Mexico | 455 | 78 (17.1) | 51 (11.2) |
|  | Panama | 92 | 2 (2.2) | 10 (10.9) |
| South-East Asia | India | 3,139 | 498 (15.9) | 98 (3.1) |
|  | Indonesia | 221 | 58 (26.2) | 37 (16.7) |
| Europe | Austria | 209 | 56 (26.8) | 50 (23.9) |
|  | Czech Republic | 454 | 106 (23.3) | 102 (22.5) |
|  | Denmark | 41 | 2 (5.4) | 4 (11.8) |
|  | France | 264 | 27 (10.6) | 64 (25.6) |
|  | Italy | 361 | 96 (26.6) | 76 (21.1) |
|  | Netherlands | 162 | 40 (24.7) | 33 (20.4) |
|  | Norway | 79 | 14 (19.4) | 13 (20.3) |
|  | Poland | 324 | 43 (13.3) | 90 (27.8) |
|  | Russia | 588 | 239 (40.6) | 326 (55.4) |
|  | Spain | 225 | 36 (16.0) | 36 (16.0) |
|  | Sweden | 236 | 45 (19.1) | 35 (15.4) |
|  | Turkey | 536 | 108 (20.1) | 86 (16.0) |
| Eastern Mediterranean | Bahrain | 70 | 16 (22.9) | 9 (12.9) |
|  | Egypt | 583 | 124 (21.3) | 55 (9.4) |
|  | Jordan | 271 | 28 (10.3) | 33 (12.2) |
|  | Kuwait | 51 | 16 (31.4) | 6 (11.8) |
|  | Lebanon | 348 | 49 (14.1) | 40 (11.5) |
|  | Oman | 31 | 10 (32.3) | 1 (3.2) |
|  | Saudi Arabia | 519 | 105 (20.2) | 51 (9.8) |
|  | Tunisia | 214 | 41 (19.2) | 18 (8.4) |
|  | UAE | 95 | 10 (10.5) | 5 (5.3) |
| Western Pacific | Australia | 167 | 52 (31.1) | 24 (14.4) |
|  | China | 1,293 | 156 (12.1) | 134 (10.4) |
|  | Japan | 1869 | 476 (25.5) | 209 (11.2) |
|  | Malaysia | 334 | 88 (26.3) | 30 (9.0) |
|  | South Korea | 236 | 23 (9.7) | 28 (11.9) |
|  | Taiwan | 258 | 23 (8.9) | 28 (10.9) |

Percentages were calculated for all patients with data available; unreported data were excluded

*UAE* United Arab Emirates


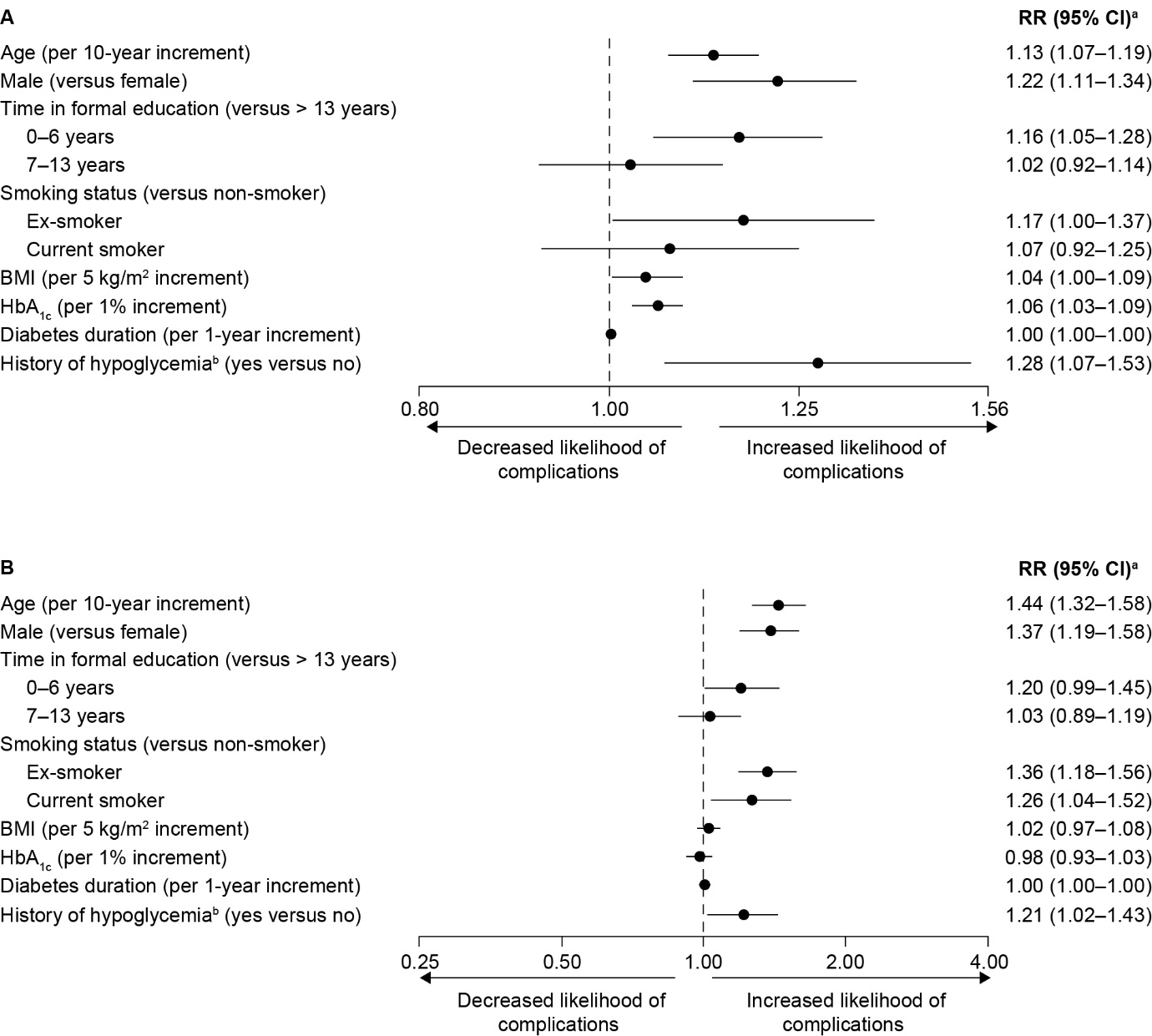


**Figure S1.** Sensitivity analysis including only patients with complete data to assess factors associated with (A) microvascular and (B) macrovascular complications. ^a^RRs adjusted for all variables in the figure with the addition of SBP, total, cholesterol levels and comedication use, using a modified Poisson model with cluster-based sandwich variance estimator as described in the Methods. RRs for the associations between complication prevalence and SBP, total cholesterol levels, and comedication use are not reported due to reverse-causality. ^b^Minor hypoglycemic event in the previous month or major hypoglycemic event in the previous year. *BMI* body mass index, *CI* confidence interval, *HbA_1c_* glycated hemoglobin, *OR* odds ratio

**
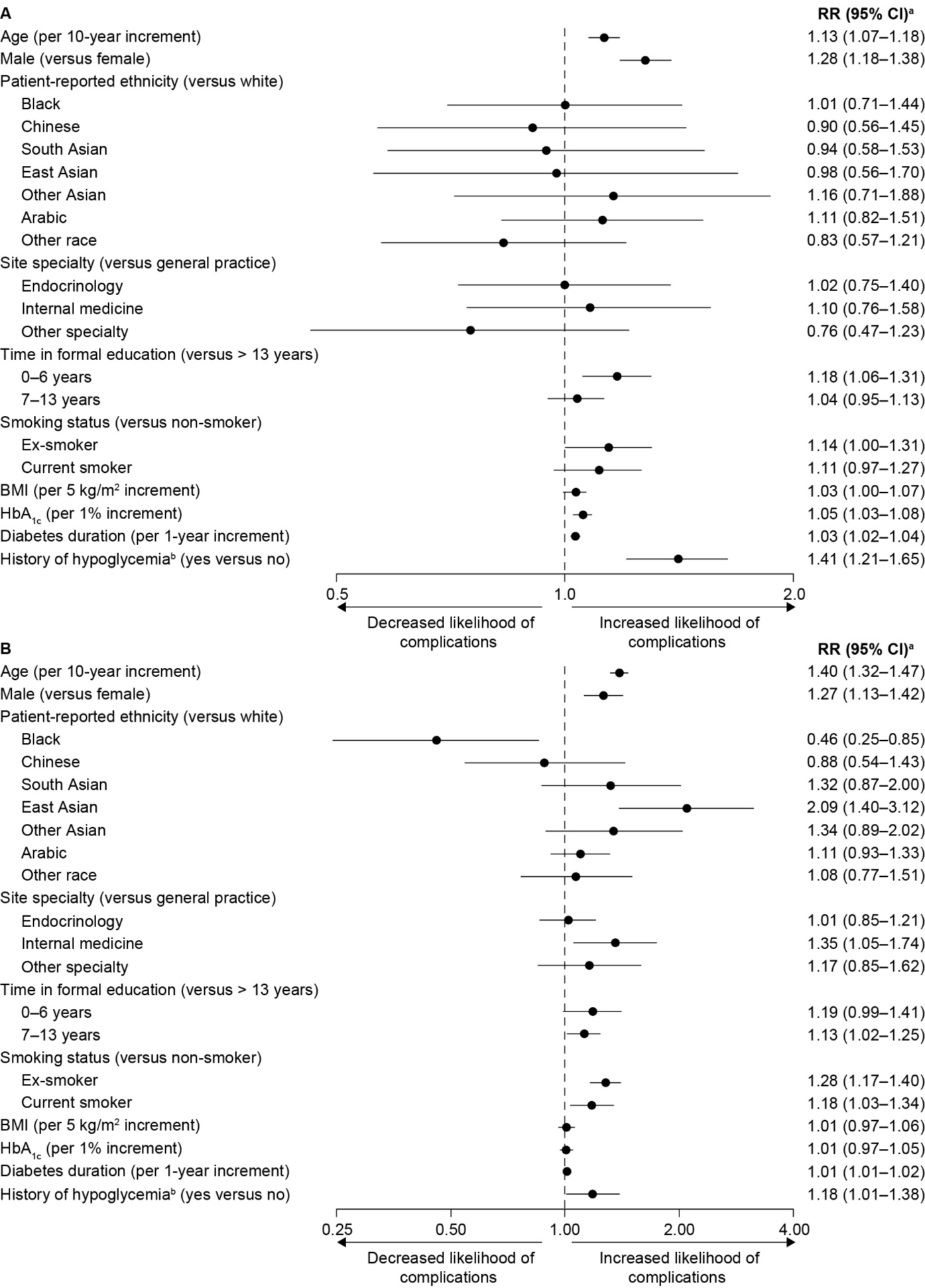
**

**Figure S2.** Sensitivity analysis with additional variables for site specialty and patient-reported ethnicity to assess factors associated with (A) microvascular and (B) macrovascular complications. ^a^RRs adjusted for all variables in the figure with the addition of SBP, total, cholesterol levels and comedication use, using a modified Poisson model with cluster-based sandwich variance estimator as described in the Methods. RRs for the associations between complication prevalence and SBP, total cholesterol levels, and comedication use are not reported due to reverse-causality. ^b^Minor hypoglycemic event in the previous month or major hypoglycemic event in the previous year. *BMI* body mass index, *CI* confidence interval, *HbA_1c_* glycated hemoglobin, *OR* odds ratio
